# Preliminary analysis of short-term real-world outcomes of telitacicept in high-risk IgA nephropathy

**DOI:** 10.3389/fmed.2025.1698028

**Published:** 2025-12-05

**Authors:** Ying Ma, Jing Han, Huixian Li, Xiao Yu, Ping Lan, Xinfang Xie, Wanhong Lu, Jiping Sun

**Affiliations:** 1 Department of Nephrology, Kidney Hospital, The First Affiliated Hospital of Xi’an Jiaotong University, Xian, China; 2 Department of Nephrology, Xi’an No.3 Hospital, The Affiliated Hospital of Northwest University, Xi’an, China; 3 Department of Nephrology, Shaanxi Provincial Hospital of Traditional Chinese Medicine, Xi’an, China

**Keywords:** IgA nephropathy, telitacicept, effectiveness, safety, real-world cohort study

## Abstract

**Introduction:**

IgA nephropathy (IgAN) represents an important cause of end-stage kidney disease worldwide. Telitacicept has demonstrated potential in attenuating disease progression in IgAN patients. However, whether its efficacy differs between initial and alternative therapy in IgAN patients at high risk of kidney function progress (high-risk IgAN) remains unclear. This preliminary real-world study seeks to provide initial insights into this question.

**Methods:**

We enrolled patients with primary IgAN who exhibited persistent proteinuria (≥ 0.75 g/day) and an estimated glomerular filtration rate (eGFR) ≥ 30 mL/min/1.73 m2 despite ≥ 3 months of supportive therapy. Participants receiving telitacicept as either initial or alternative treatment were propensity score-matched (1:1) based on baseline proteinuria and eGFR. A control group initiating conventional immunosuppressants (initial IS group) was included for comparison. Effectiveness endpoints included the renal response (RR) rate, defined as a complete (proteinuria < 0.5 g/day) or partial (>50% reduction and < 1 g/day in proteinuria) response, with both requiring stable renal function (eGFR decline ≤30%), as well as changes in proteinuria and eGFR from baseline during follow-up.

**Results:**

A total of 138 patients were included in the full study cohort. After propensity score matching, ninety participants constituted the matched cohort, comprising 30patients in each of the three groups. At 3 months, the initial telitacicept group showed a median proteinuria reduction of 1.47 g/day (79% from baseline), comparable to the initial IS group (1.15 g/day, 48%) but significantly greater than the alternative telitacicept group (0.88 g/d, 46%). Concurrently, eGFR remained stable. 25 patients (83.3%) in the initial telitacicept group achieved RR-a rate significantly higher than in the other two groups. At 6 months, proteinuria in the initial telitacicept group continued to decline to 0.47 (0.20, 1.22) g/day, a level comparable to the initial IS group and numerically lower than the alternative telitacicept group. Throughout the follow-up, eGFR remained stable in the initial telitacicept group, whereas it exhibited greater fluctuation in the initial IS group. No serious adverse events were reported.

**Conclusion:**

Our preliminary real-world findings suggest that telitacicept may be a safe and effective treatment for high-risk IgAN patients, significantly reducing proteinuria and preserving renal function. Its therapeutic benefits were more pronounced when used as initial therapy.

## Introduction

IgA nephropathy (IgAN), the most common primary glomerular disease worldwide, remains one of the leading causes of kidney failure (1–3), with higher prevalence (40–50%) and worse prognosis in Asian populations. The current risk stratification defines high-risk IgAN as sustained proteinuria (>0.75–1 g/day) despite ≥ 90 days of optimized supportive care, a key indicator for progressive kidney function loss necessitating intervention (2). Herein, maximal supportive therapies incorporating immunosuppressive drugs should be considered (2), with glucocorticoids remain the main therapy to patients from high-risk population (4). Nevertheless, recent prospective data from a Chinese IgAN cohort reveal an alarming annual event rate of renal failure is as high as 41.1 per 1,000 patient-years, and a median kidney survival time of only about 10 years (5). These findings underscore the inadequacy of existing therapies in controlling disease progression and highlight the potential benefits of developing new treatments. Additionally, a subset of IgAN patients exhibits refractoriness to conventional immunosuppressive treatment, warranting alternative therapeutic strategies (6).

IgAN is dirven by mesangial IgA1 deposition, with the prevailing ‘multi-hit’ hypothesis identifying galactose-deficient IgA1 (Gd-IgA1) produced by B cells as first hit (3, 7). In most IgAN patients, elevated circulating levels of Gd-IgA1 and its corresponding antibodies are associated with an increased risk of disease progression (4, 7). Notably, the B cell growth cytokines BAFF (B cell-activating factor) and APRIL (a proliferation-inducing ligand) play pivotal roles in B cell proliferation, differentiation and mutation, as well as IgA class switching (3). Moreover, BAFF reportedly promotes human mesangial cell proliferation (8), and elevated APRIL levels in IgAN are associated with more severe clinical manifestations and poor prognosis (9). Consequently, inhibition of BAFF/APRIL represents a promising therapeutic avenue for IgAN.

Telitacicept is a novel fusion protein that competitively binds BAFF/APRIL via the extracellular domain of transmembrane activator and calcium-modulating cyclophilin ligand interactor (TACI), thereby suppressing aberrant B cell activation (10). To date, phase 2 clinical trial in IgAN patients with persistent proteinuria have demonstrated the efficacy and safety of telitacicept in reducing proteinuria (4), with real-world studies confirming rapid benefits (34.5–75.7% reduction in proteinuria from baseline over 2–6 months) (11–13), as well as long-term benefits (proteinuria reduction from 1.6 g/day to 0.6 g/day at 12 months, alongside improved eGFR) (14). Previous multi-center retrospective study, which involved a substantial proportion (65%) of patients switching to telitacicept due to inadequate response to their prior treatment, similarly reported significant proteinuria reduction (11). It indicated that telitacicept may serve as an effective alternative for IgAN patients refractory to conventional IS treatments (11). And the latest Chinese clinical practice guidelines (2025) have suggested that telitacicept can be considered as a new treatment option for high risk IgAN or refractory IgAN. Despite this guideline update, telitacicept has not yet been covered by national medical insurance, requiring full out-of-pocket payment. While telitacicept is increasingly used as either initial therapy or an alternative to conventional immunosuppressants for IgAN, comparative data on its efficacy in these distinct clinical settings remain limited. Therefore, the present preliminary study directly addresses this gap by evaluating the performance of telitacicept as initial therapy versus alternative therapy in high-risk IgAN patients in real world.

## Materials and methods

### Study population

**Figure 1:**
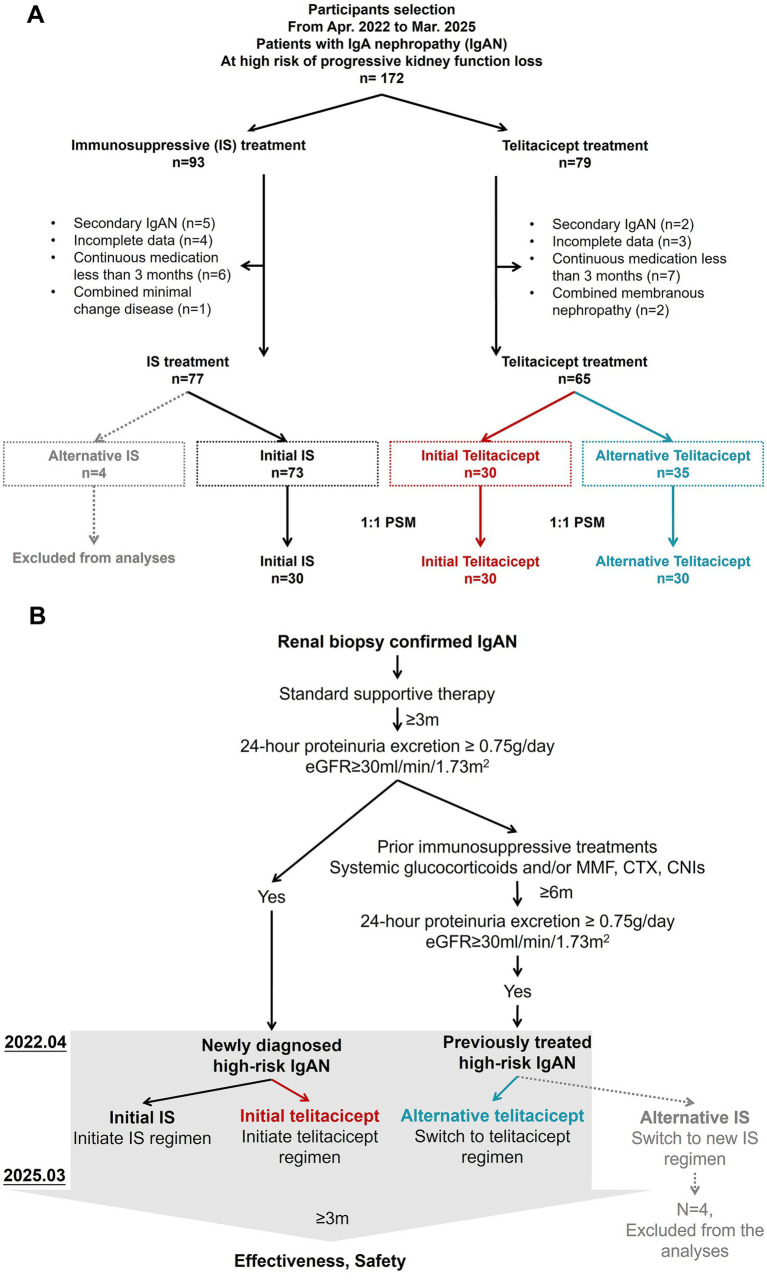
Participant flowchart and therapeutic trajectories. **(A)** Recruitment and exclusion flowchart of the cohort. Separate propensity score match (PSM) for the initial telitacicept group against both the initial IS group and the alternative telitacicept group was performed on the basis of baseline proteinuria and eGFR level. Matching was performed using 1:1 nearest-neighbor matching without replacement, with a caliper width of 0.02. **(B)** Therapeutic trajectories of the cohorts. MMF, mycophenolate mofetil; CTX, cyclophosphamide; CNIs, calcineurin inhibitors; IS, conventional immunosuppressants including glucocorticoids and/or MMF, CTX, CNIs. Alternative IS group was excluded from the final cohort analyses due to the small size.

This single-center, retrospective study enrolled IgAN patients at high risk of kidney function loss who received immunosuppressive therapy at Nephrology Department of the First Affiliated Hospital of Xi’an Jiaotong University, a large, comprehensive Class A tertiary hospital and a major national medical center in Northwest China, between April 2022 and March 2025. Figure 1 illustrated the study flowchart and participant therapeutic trajectories. The key Inclusion criteria were as follow: (1) age ≥ 18 years at baseline; (2) high-risk IgAN patients, IgAN patients at high risk of progression (i.e., high-risk IgAN), which defined as biopsy-confirmed IgAN patients sustained proteinuria (24-h proteinuria excretion ≥ 0.75 g/day) despite ≥ 3 months of standard supportive treatments (2, 4). Supportive treatment referred to a maximum or tolerated dose of renin angiotensin-aldosterone system inhibitors (RAASi). (3) estimated glomerular filtration rate (eGFR) ≥ 30 mL/min/1.73m^2^; and (4) receiving telitacicept [subcutaneous injection of 160 mg or 240 mg once a week referring to the dosage in clinical trial (4)] or conventional immunosuppressants (IS) including glucocorticoids and/or mycophenolate mofetil (MMF), cyclophosphamide (CTX), and calcineurin inhibitors (CNIs). We excluded patients with (1) secondary IgAN such as IgA vasculitis, lupus nephritis, or inflammatory bowel disease-associated IgAN; (2) comorbid conditions affecting treatment such as pathology confirming concurrent membranous nephropathy, minimal change disease or diabetic nephropathy; (3) continuous treatment duration < 3 months; (4) incomplete baseline or follow-up data; and (5) history of plasma exchange or other B cell target therapy (e.g., rituximab) with 3 months.

### Patient groups

As shown in Figures 1A,B, patients were stratified into two treatment groups based on their primary immunosuppressive regimen in our center. Those received conventional IS were in IS group, whereas those received telitacicept were in telitacicept group. Subclassification was further performed according to the previous treatment history. Those newly diagnosed high-risk IgAN patients without prior immunosuppressive therapy were classified into initial treatment groups, and those who switched to a new regimen after prior immunosuppressive treatment were classified into alternative treatment groups.

### Data collection

The baseline time was defined as the date of telitacicept / IS initiation. Baseline laboratory variables were collected from the single value obtained closest to this index date, within a measurement window of 1 months prior to treatment initiation. All clinical characteristics for matching and baseline comparisons were anchored to this definition. Regular outpatient visits were managed by IgAN specialty team of our center. Baseline and follow-up data were collected, including demographics variables such as age, gender, body mass index (BMI), and blood pressure, as well as laboratory parameters including 24-h proteinuria excretion, hematuria, serum albumin, eGFR (calculated by the 2021 CKD–EPI formula), serum immunoglobulins (IgA, IgM, IgG). Renal pathologic data, including Oxford classification MEST-C scores (mesangial [M] and endocapillary [E] proliferation (hypercellularity), segmental glomerulosclerosis [S], tubular atrophy / interstitial fibrosis [T], and crescents [C]) were collected. Concomitant medications including RAASi, sodium-glucose cotransporter 2 inhibitors (SGLT2i), and hydroxychloroquine (HCQ) were recorded. Adverse events (AEs) were regularly reported. Discontinuation was defined as an interruption of telitacicept or IS treatments for more than 3 months. Data collection was ceased at medication discontinuation.

### End points

The primary effectiveness endpoints were the renal response (RR) at 3 months of treatment, referring to complete renal response (CRR) or partial renal response (PRR). Precisely, CRR was defined as proteinuria < 0.5 g/day and stable renal function (eGFR decline ≤ 30%) (15, 16). Proteinuria reduction >50% reduction and < 1 g/day in 24-h proteinuria while maintaining stable renal function without achieving CRR were classified as PRR (12, 14). The secondary efficacy endpoints were absolute or percentage changes in proteinuria and eGFR from baseline, as well as renal response rate at follow-up intervals.

The safety endpoints was the incidence of AEs, such as injection site reactions, infections (e.g., respiratory tract infection, urinary tract infection, and herpes virus infection), liver dysfunction, and metabolic disorders including hyperglycemia, hyperuricemia, osteoporosis, and skin acne. Severe AEs (SAEs) was defined as AEs related with hospitalization or death.

### Statistical analyses

Categorical variables were expressed as frequencies and percentages and were compared with chi-square (χ2) test. Normally distributed continuous variables were presented as mean ± standard deviation and were analyzed by Student’s t test. Nonnormally distributed continuous variables were described as median values with interval quartile ranges (IQR) and were analyzed with Mann–Whitney U test. Generalized estimating equations (GEE) were built to compare time-series data of each group and analyze change tendency of variables over time. Analyses were performed by R software (version 4.4.3, R Foundation for Statistical Computing, Vienna, Austria) and R packages (such as compareGroups and geeglm). A two-sided *p*-value of < 0.05 was considered statistically significant.

We perform separate propensity score match (PSM) for the initial telitacicept group against both the initial IS group and the alternative telitacicept group on the basis of baseline proteinuria and eGFR level, as these are the only prognostic biomarkers for IgAN endorsed by the lasted KDIGO guidelines (15). Matching was performed using 1:1 nearest-neighbor matching without replacement, with a caliper width of 0.02 standard deviations of the logit of the propensity score (Supplementary Table 1). Standardized Mean Differences (SMDs) were used to assess inter-group differences, with SMD1 for the comparison between the initial telitacicept and initial IS groups, and SMD2 for that between the initial telitacicept and alternative telitacicept groups. An SMD < 0.1 was considered to indicate a good balance. Additionally, we performed inverse probability of treatment Weighting (IPTW) based on the propensity scores for robustness analysis.

## Results

### Baseline data of IgAN patients at progressive kidney function loss

A total of 172 high-risk IgAN patients were identified at our center between April 2022 and March 2025. After applying the exclusion criteria, 138 patients were included in the full study cohort (Figure 1A). This cohort comprised 103 newly diagnosed patients, of whom 73 received conventional immunosuppressants (initial IS group) and 30 received telitacicept as initial therapy (initial telitacicept group). The remaining 35 patients, who had a prior immunosuppressant treatment history, were switched to telitacicept (alternative telitacicept group). Thus, a total of 65 patients received telitacicept (the initial and alternative telitacicept groups combined), none of whom were participants in a commercial clinical trial for the drug. The baseline characteristics of all patients are shown in Table 1.

**Table 1:** Balance of covariates and propensity score before and after matching.

Characteristics	Before matching (full cohort)					After matching (matched cohort)			
	Initial telitacicept *N* = 30	Initial IS *N* = 73	Alternative telitacicept *N* = 35	SMD1	SMD2	Initial IS *N* = 30	Alternative telitacicept *N* = 30	SMD1	SMD2
Age, years	38.0 (33.3, 45.0)	38.0 (27.0, 47.5)	35.0 (29.0, 42.0)	0.135	0.294	38.0 (31.0, 47.0)	34.5 (28.8, 41.0)	0.040	0.356
Male, *n* (%)	17 (56.7)	40 (54.8)	20 (57.1)	0.077	0.030	17 (56.7)	15 (50.0)	<0.001	0.134
BMI, kg/m^2^	24.9 ± 4.1	24.4 ± 3.9	25.4 ± 6.2	0.134	0.089	24.8 ± 4.1	24.6 ± 4.1	0.039	0.080
SBP, mmHg	127.0 (113.8, 138.8)	122.0 (111.5, 137.0)	128.0 (111.0, 137.0)	0.092	0.094	122.5 (113.0, 135.5)	125.0 (109.8, 137.0)	0.113	0.089
DBP, mmHg	82.5 ± 14.0	80.1 ± 13.5	83.4 ± 13.4	0.124	0.066	83.3 ± 12.9	83.2 ± 13.4	0.057	0.054
Medical history									
Hypertension	15 (50.0)	23 (31.5)	14 (40.0)	0.383	0.202	10 (33.3)	11 (36.7)	0.343	0.272
Diabetes mellitus	6 (20.0)	10 (13.7)	3 (8.6)	0.169	0.331	4 (13.3)	2 (6.7)	0.180	0.400
Time since diagnostic kidney biopsy, months	3.7 (3.2, 4.5)	3.3 (3.2, 3.8)	44.3 (7.8, 86.0)	0.341	1.111	3.5 (3.2, 3.8)	43.7 (7.9, 86.3)	0.329	1.108
Oxford classification, *n* (%)									
M1	17 (56.7)	34 (46.6)	27 (77.1)	0.203	0.446	2 4 (80.0)	23 (76.7)	0.518	0.434
E1	9 (30.0)	28 (38.4)	10 (28.6)	0.301	0.287	13 (43.3)	8 (26.7)	0.279	0.074
S1	24 (80.0)	56 (76.7)	28 (80.0)	0.080	<0.001	24 (80.0)	23 (76.7)	<0.001	0.081
T1/2	10 (33.3)	16 (21.9)	9 (25.7)	0.257	0.168	4 (13.3)	7 (23.3)	0.487	0.223
C1/2	11 (36.7)	30 (41.1)	12 (34.3)	0.091	0.050	9 (30.0)	10 (33.3)	0.142	0.070
Hemoglobin, g/L	129.4 ± 24.1	129.0 ± 20.2	133.6 ± 22.1	0.017	0.184	130.1 ± 22.1	131.6 ± 21.7	0.030	0.095
Serum albumin, g/L	37.4 (29.1, 39.0)	33.8 (29.5, 39.7)	37.0 (31.1, 40.3)	0.089	0.221	33.7 (30.8, 39.9)	36.0 (31.0, 39.7)	0.119	0.145
eGFR, ml/min/1.73m^2^	76.6 ± 31.5	83.9 ± 33.2	73.2 ± 33.7	0.227	0.103	75.3 ± 27.8	77.6 ± 34.1	0.044	0.032
Proteinuria, g/day	2.04 (1.60, 4.16)	2.15 (1.34, 3.37)	1.96 (1.23, 3.19)	0.029	0.071	2.01 (1.34, 3.06)	1.99 (1.32, 3.75)	0.001	0.012
Microscopic hematuria, RBCs/HP	14.9 (5.7, 47.5)	30.2 (11.0, 77.7)	8.3 (4.8, 23.0)	0.164	0.541	26.8 (10.3, 57.7)	9.1 (4.3, 23.5)	0.105	0.518
Serum IgA, g/L	2.9 ± 0.8	3.4 ± 1.2	2.7 ± 1.4	0.192	0.208	2.7 ± 0.8	2.5 ± 0.8	0.262	0.621
Serum IgG, g/L	8.9 ± 2.7	8.8 ± 3.4	9.0 ± 3.7	0.032	0.027	8.7 ± 3.8	8.8 ± 3.3	0.061	0.040
Serum IgM, g/L	0.8 (0.5, 1.3)	0.9 (0.6, 1.2)	0.8 (0.6, 1.0)	0.139	0.172	0.8 (0.6, 1.1)	0.8 (0.7, 1.2)	0.145	0.052
Concurrent glucocorticoids, *n* (%)	10 (33.3)	70 (95.9)	11 (31.4)	1.730	0.041	30 (100.0)	9 (30.0)	2.000	0.072
Concomitant therapy									
RAASi, *n* (%)	27 (90.0)	65 (89.0)	31 (88.6)	0.031	0.046	29 (96.7)	26 (86.7)	0.270	0.104
SGLT2i, *n* (%)	16 (53.3)	35 (47.9)	18 (51.4)	0.108	0.038	16 (53.3)	16 (53.3)	0.134	<0.001

The Standardized Mean Differences (SMDs) were used to assess inter-group differences, and an SMD < 0.1 was considered to indicate a good balance. SMD1: SMD between the initial telitacicept group and the initial IS group; SMD2: SMD between the initial telitacicept group and the alternative telitacicept group. BMI, body mass index; SBP, systolic blood pressure; DBP, diastolic blood pressure; M, mesangial hypercellularity (M0, < 50% of glomeruli show mesangial hypercellularity; M1, > 50% of glomeruli show mesangial hypercellularity); E, endocapillary hypercellularity (E0, no endocapillary hypercellularity; E1, any glomeruli show endocapillary hypercellularity); S, segmental glomerulosclerosis (S0, absent, S1, present in any glomeruli); T, tubular atrophy/interstitial fibrosis (T0, 0–25% of cortical area; T1, 26–50% of cortical area; T2, > 50% of cortical area); C, crescents (C0, absent; C1, 0–25% of glomeruli; C2, ≥25% of glomeruli); eGFR, estimated glomerular filtration rate, calculated by the 2021 Chronic Kidney Disease Epidemiology Collaboration formula. RAASi, renin–angiotensin–aldosterone system inhibitor; SGLT2i, sodium–glucose co-transporter 2 inhibitor.

After performing 1:1 PSM, we enrolled 90 patients in the final analyses (Figure 1A). There were no missing data for the propensity model covariates (baseline proteinuria and eGFR) thus no imputation was required. As shown in Table 1, the matching successfully achieved excellent balance (all SMDs <0.1) for the specifically targeted covariates-baseline proteinuria and eGFR-as intended. We also observed improved balance (i.e., a reduction in SMD) for several non-matched variables, such as age, gender, BMI (see SMD1 in Table 1). However, some non-matched variables, including medical history, time since diagnostic kidney biopsy, concomitant therapy, remained imbalance after matching (SMDs˃0.1).

To address residual confounding in some covariates after PSM, we performed an adjusted logistic analysis on the matched cohort. Given the sample size of the matched cohort, we prioritized adjustment for clinically established strong prognostic factors for IgAN progression, which included baseline proteinuria, eGFR, and the use of RAASi and SGLT2i. The telitacicept treatment effect estimate from this adjusted logistic model was consistent with our primary unadjusted analysis (Supplementary Table 2). Alternative sensitivity analyses using IPTW yielded similar results (Supplementary Figures 1, 2). The consistency of these findings across different analytical approaches supports the robustness of our primary conclusion.

The study population comprised 30 newly diagnosed high-risk IgAN patients who initiated telitacicept as initial therapy (initial telitacicept group), exhibiting median proteinuria level of 2.04(1.60, 4.16) g/day and mean eGFR of 76.6 ± 31.5 mL/min/1.73m^2^. Through 1:1 PSM based on baseline proteinuria and eGFR level, we selected 30 comparable patients receiving conventional IS as initial therapy (initial IS group) (Figure 1A). Both groups showed balanced baseline characteristics including age, gender, BMI, and hemoglobin (Table 1, SMD1<0.1), whereas some characteristics exhibited imbalance at baseline (e.g., concurrent glucocorticoids). In the initial telitacicept group, 10 patients (33.3%) received adjunctive oral glucocorticoids at a median dose of 30.0 mg/day (IQR 23.8–30.0, prednisone-equivalent), while all patients in the initial IS group received oral glucocorticoids (median dose 30.0 mg/day, IQR 28.8–36.3, prednisone-equivalent), with 11 patients (36.7%) receiving additional immunosuppressants, predominantly CTX (*n* = 9, 81.8%).

There were 35 high-risk IgAN patients with previous immunosuppressive treatment exposure who switched to telitacicept as alternative therapy due to disease refractoriness (51.4%) or flare (48.6%). After 1:1 PSM with initial telitacicept group, 30 patients were selected for the alternative telitacicept group (Figure 1A). These patients had significantly longer median time duration since diagnostic kidney biopsy (43.7 months, IQR 7.9–86.3) compared to the initial telitacicept group (3.7 months, IQR 3.2–4.5) (*p* < 0.001). Previous immunosuppressive treatments predominantly involved glucocorticoids (*n* = 29, 96.7%), followed by CTX (*n* = 9, 30%) and MMF (*n* = 8, 26.7%), and with minimum treatment duration of 3.5 months. Telitacicept dosing patterns were similar between groups, with 86.7% of alternative therapy patients receiving 160 mg weekly versus 93.3% in the initial therapy group (*p* = 0.671).

### Effectiveness

#### Effects on renal parameters

**Figure 2:**
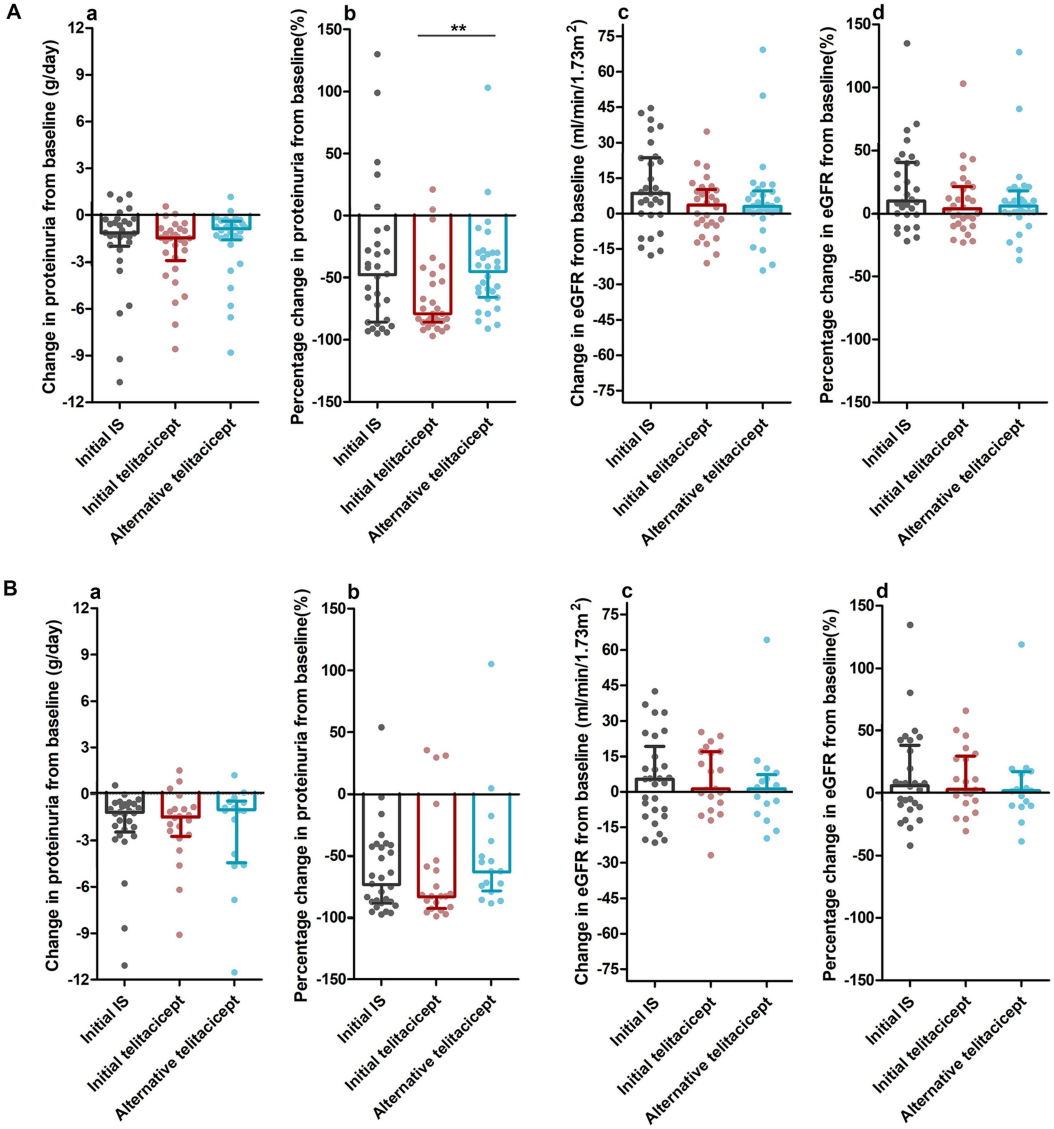
Changes in proteinuria and eGFR levels from baseline during follow-up. Data are expressed as medians and interquartile ranges. **(A)** At 3 months, **(B)** at 6 months. **(a)** Absolute change in proteinuria from baseline; **(b)** percentage change in proteinuria from baseline; **(c)** absolute change in eGFR from baseline; **(d)** percentage change in eGFR from baseline. **p < 0.01.

The initial telitacicept group demonstrated significant reductions in proteinuria, decreasing from a median baseline level of 2.04 g/day (IQR 1.60–4.16) to 0.58 g/day (IQR 0.31–1.04) at 3 months, representing a median absolute reduction of 1.47 g/day (79%, *p* < 0.001). As shown in Figure 2Aa, comparable absolute reductions were observed in the initial IS group (1.15 g/day, *p* < 0.001) and the alternative telitacicept group (0.88 g/day, *p* < 0.001). The percentage reduction of proteinuria in the initial telitacicept group was similar to that in the initial IS group (*p* = 0.065, Figure 2Ab), whereas significantly greater than that in the alternative telitacicept group (*p* = 0.002, Figure 2Ab).

Renal functions, as measured by eGFR, showed modest changes across groups. The initial telitacicept group exhibited a nonsignificant increase in eGFR from 76.6 ± 31.5 mL/min/1.73m^2^ at baseline to 79.3 ± 28.3 mL/min/1.73m^2^ at 3 months, reaching an absolute increase by 3.6 mL/min/1.73m^2^ (4% increase, *p* = 0.232), while the initial IS group demonstrated a more pronounced improvement (8.5 mL/min/1.73m^2^, 10% increase, p = 0.002). However, between-group comparisons revealed no significant differences in either absolute change or percentage eGFR changes (*p* = 0.088 and *p* = 0.141, respectively, Figures 2Ac,d). Meanwhile, the alternative telitacicept group showed a slight eGFR change by 3.1 mL/min/1.73m^2^ (6% increase, *p* = 0.106) at the third month, with no significant differences compared to the initial telitacicept group (Figure 2Ac,d).

Among the 60 patients treated with telitacicept, 37 (61.7%) remained on treatment for over 6 months. Treatment discontinuations occurred for the following reasons: 39.1% (9/23) patients discontinued as permitted by the study protocol after CRR or PRR; 34.8% (8/23) patients withdrew due to financial burden (lack of national medical insurance coverage in China); and 26.1% (6/23) due to insufficient treatment duration at the data cutoff. At the 6-month follow-up, the initial telitacicept group achieved a median reduction in proteinuria of 1.54 g/d (82.8%) from baseline, resulting in a median value of 0.47 g/d. Although this reduction was numerically greater than in the initial IS and alternative telitacicept groups, the differences were not statistically significant (Figure 2Ba,b). Meanwhile, the eGFR in the initial telitacicept group was maintained at a level comparable to the other two groups (Figure 2Bc,d). Detailed data on changes in renal parameters during the follow-up period are provided in Supplementary Table 3.

**Figure 3:**
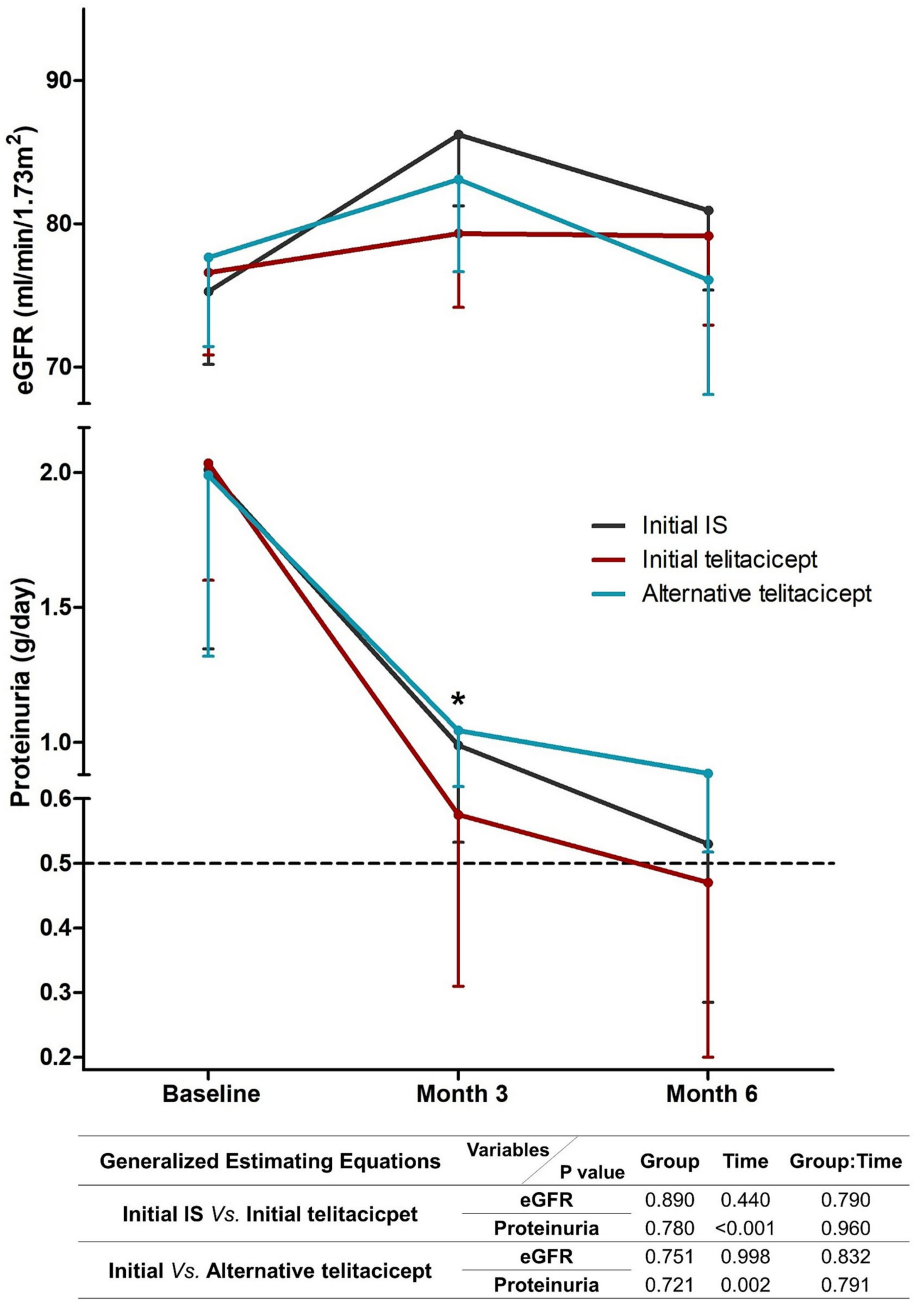
Change tendency of proteinuria and eGFR of the 3 groups during follow-up period. *Initial telitacicept group versus alternative telitacicept group, *p* < 0.05.

Longitudinal analysis revealed continued proteinuria reduction across all groups during the 6-month follow-up, with GEE analysis confirming significant temporal trends (*p* < 0.05) but no between-group differences in the decline tendency (Figure 3). The eGFR trajectories differed slightly among groups: while stable in the initial telitacicept group, the initial IS and the alternative telitacicept groups showed early improvement followed by late decline. However, GEE analysis detected no significant differences in eGFR trends either over time or between treatment groups (Figure 3).

#### Treatment response

Prior to PSM matching, analysis of the full unmatched cohort at 3 months revealed that the initial telitacicept group achieved significantly higher rates of both CRR (46.7% versus 24.7%, *p* = 0.028) and overall RR (83.3% versus 60.3%, *p* = 0.024) compared to the initial IS group. Furthermore, when compared to the alternative telitacicept group, the initial telitacicept group demonstrated a significantly greater CRR rate (46.7% versus 14.3%, *p* = 0.004), although the difference in overall RR was not statistically significant (83.3% versus 62.9%, *p* = 0.066).

After matching, the landscape of comparisons at 3 months evolved (Table 2). The CRR advantage of the initial telitacicept group over the initial IS group was attenuated and no longer statistically significant (46.7% versus 23.3%, *p* = 0.058), while its superiority over the alternative group in CRR remained robust (46.7% versus 14.3%, *p* = 0.005). The overall RR rate in the initial telitacicept group (83.3%) was significantly higher than that of both matched groups, as evidenced by significantly more responders compared to the initial IS (83.3% vs. 56.7%, *p* = 0.024) and alternative telitacicept groups (83.3% vs. 60.0%, *p* = 0.045).

**Table 2:** Treatment response of the 3 groups.

	Initial IS	Initial telitacicept	Alternative telitacicept	*p*1 value	*p*2 value
Month 3	*N* = 30	*N* = 30	*N* = 30		
CRR, *n* (%)	7 (23.3)	14 (46.7)	4 (13.3)	0.058	0.005
PRR, *n* (%)	10 (33.3)	11 (36.7)	14 (46.7)	0.787	0.432
RR, *n* (%)	17 (56.7)	25 (83.3)	18 (60.0)	0.024	0.045
Month 6	*N* = 29	*N* = 21	*N* = 16		
CRR, *n* (%)	14 (48.3)	11 (52.4)	3 (18.8)	0.774	0.037
PRR, *n* (%)	5 (17.2)	6 (28.6)	9 (56.3)	0.342	0.089
RR, *n* (%)	19 (65.5)	17 (81.0)	12 (75.0)	0.230	0.705

*p*1 value: the initial telitacicept group versus the initial IS group; *p*2 value: the initial telitacicept group versus the alternative telitacicept group. CRR, compete renal response; PRR, partial renal response; RR, renal response (CRR or PRR).

By the 6-month follow-up in the matched cohort, the pattern of responses continued to evolve (Table 2). The CRR rate in the initial telitacicept group increased to 52.4%, which was now comparable to the initial IS group (48.3%, *p* = 0.774) but remained significantly higher than the alternative telitacicept group (18.8%, *p* = 0.037). Accompanying these changes, PRR rate declined to 28.6% and 17.2% in the initial telitacicept group and the initial IS group, respectively. Conversely, PRR increased to 56.3% in the alternative telitacicept group. There were no significant differences in final RR rates between the initial telitacicept group and the other two groups.

### Safety

The AEs profiles across the three groups are detailed in Table 3. No SAEs were reported during the study period. The initial telitacicept group demonstrated a favorable safety profile, with only 5 AEs (16.7%) reported, the majority of which were infections (*n* = 4). The overall AEs incidence in the initial telitacicept group was significantly lower than that in the initial IS group (*p* = 0.006) but comparable to the alternative telitacicept group. Metabolic disorders, including hyperglycemia, hyperuricemia, osteoporosis and kin acne, were exclusively observed in the initial IS group, with significantly higher incidence compared to the initial telitacicept group (*p* = 0.026, Table 3). Injection site reactions, a characteristic AE associated with subcutaneous administration, were uniquely reported in telitacicept-treated groups, and with no statistic difference in frequency between the initial and alternative group.

**Table 3:** Adverse events in the 3 groups.

AEs	Initial IS *N* = 30	Initial telitacicept *N* = 30	Alternative telitacicept *N* = 30	*p*1 value	*p*2 value
AEs in total	15 (50.0)	5 (16.7)	6 (20.0)	0.006	0.739
Injection site reactions	0 (0.0)	1 (3.3)	3 (10.0)	0.500	0.612
Infections	9 (30.0)	4 (13.3)	3 (10.0)	0.117	0.687
Respiratory tract infection	6 (20.0)	3 (10.0)	2 (6.7)		
Urinary system infection	2 (6.7)	1 (3.3)	1 (3.3)		
Herpes virus infection	1 (3.3)	0 (0.0)	0 (0.0)		
Metabolic disorders	5 (16.7)	0 (0.0)	0 (0.0)	0.026	n/a
Liver dysfunction	1 (3.3)	0 (0.0)	0 (0.0)	0.500	n/a

*p*1 value: the initial telitacicept group versus the initial IS group; *p*2 value: the initial telitacicept group versus the alternative telitacicept group. n/a, not available.

## Discussion

This real-world, observational study evaluated the effectiveness and safety profile of telitacicept in IgAN patients at high risk of kidney function loss (high-risk IgAN). The findings demonstrated that high-risk IgAN patients initially treated by telitacicept achieved superior clinical outcomes compared to those as alternative treatment after previous IS exposure. Compared to the alternative telitacicept group, the initial telitacicept group exhibited significantly greater reduction in proteinuria (median 79% decrease from baseline) while maintaining stable renal function. Furthermore, this group attained higher renal response rate (83.3% at 3 months) and exhibited a more favorable safety profile, with fewer overall AE incidence compared to initial IS group.

Our study supported the effectiveness of telitacicept in high-risk IgAN patients. The therapeutic potential of telitacicept in IgAN stems from its dual inhibition of BAFF and APRIL, two cytokines that play pivotal roles in IgAN pathogenesis (17, 18). By targeting these key mediators, telitacicept offers a mechanistically grounded approach to attenuating renal progression in IgAN. Although multiple prognostic tools have been developed to assist in predicting kidney progression, including the incorporation of MEST-C score (19) and machine-learning model (20), the only validated short-term, modifiable prognostic biomarker for IgAN is proteinuria (21, 22). A reduction to < 0.5–1.0 g/day is considered a reasonable treatment target for proteinuria (2, 5, 23). In the pooled cohort of telitacicept-treated patients (combined initial and alternative groups), treatment led to a significant reduction in proteinuria, with a median decrease of 1.30 g/day (61%; *p* < 0.001) from baseline to a median of 0.86 g/day at 3 months. Additionally, a significant improvement in renal function was observed, as indicated by a median absolute increase in eGFR of 3.1 mL/min/1.73m^2^ (a 5% increase; *p* = 0.038). These robust treatment effects mirror observations from both clinical trial (4) and real-world studies (11–14). Although the phase 2 trial demonstrated that 240 mg per week, rather than 160 mg weekly, could lead to significant reduction of proteinuria, our real-world cases achieved comparable efficacy with 160 mg weekly in most patients (*n* = 50, 83.3%). A similar dosage preference and treatment efficacy was observed in other three retrospective studies of IgAN patients (11, 12, 14). The discrepancy likely reflects several clinically relevant factors. First, telitacicept cost considerations (telitacicept is not covered by national medical insurance for the treatment of IgAN in China and requires full out-of-pocket payment). Second, the existing dosing recommendations with 160 mg weekly in systemic lupus erythematosus (24) may influence prescribing patterns. Third, the frequent concomitant use of glucocorticoids [31.7–51.5% of cases (11, 12), including our study, or even 100% of cases (14)] may enhance treatment response at lower telitacicept dose, as glucocorticoids provide complementary anti-inflammatory effects (2) that help control glomerular inflammation and prevent against renal deterioration (25, 26). Finally, differences in study design, including sample size and follow-up duration, may contribute to observed effectiveness variations between trial and real-world settings.

Our study represents one of the first investigations comparing the effectiveness of telitacicept as initial versus alternative therapy in high-risk IgAN patients. This distinction carries significant clinical relevance, as there exist some IgAN patients demonstrate suboptimal response to comprehensive supportive care and immunosuppressive regimens (6, 27), creating an urgent need for targeted therapeutic alternatives. Previous multi-center retrospective study, which involved a rather high percentage of patients (65%) who were unsatisfactory or refractory to conventional IS treatments, demonstrated a significant proteinuria reduction effect of telitacicept treatment (11). It indicated that telitacicept may be an alternative therapy for IgAN patients with previous exposure to conventional IS treatments (11). However, crucial gaps remained regarding its performance as initial treatment versus as alternative therapy. In our study, thirty IgAN patients with previous IS treatments and switched to telitacicept were enrolled as the alternative telitacicept group. Among them 16 (53.3%) patients responded inadequately to the conventional IS treatments, and 14 (46.7%) patients experienced ≥ 2 times of disease flare. The median time duration since diagnostic kidney biopsy in the alternative group was as long as 43.7(7.9,86.3) months. Their median proteinuria level decreased significantly from 1.99 g/day at baseline to 1.00 g/day at 3 months (*p* < 0.001), with further decline to 0.89 g/day at 6 months, while maintaining stable eGFR. These findings support telitacicept as alternative therapy might benefit IgAN patients who were refractory or unsatisfactory to conventional treatments. And we speculated the additional effects of telitacicept as an alternative therapy might be ascribed to its different therapeutic target from conventional immunosuppressants (11). In addition, compared to the initial telitacicept group, the alternative telitacicept group showed attenuated proteinuria reduction percentage (46% versus 79%, *p* = 0.002), accompanied by a significantly lower early response rates (CRR 13.3% versus 46.7% at 3 months, *p* = 0.037). These disparities may reflect the well-documented negative prognostic impact of previous renal flare and exposure to immunosuppressive treatments (28, 29) in immune mediated glomerular diseases. However, the GEE analyses showed that 6-month change tendency of proteinuria and eGFR were comparable between the alternative and the initial telitacicept groups, with equivalent final RR rates. These findings preliminarily supported long-term use of telitacicept in high-risk IgAN patients with previous IS exposure, though it requires validation in larger prospective studies before generalization.

Telitacicept exhibited as efficiently as conventional immunosuppressants in newly diagnosed high-risk IgAN patients. Telitacicept demonstrated non-inferiority to conventional IS regimens with numerical superiority in proteinuria reduction (79% vs. 48%), corroborating Wang et al.’s findings (12). Meanwhile, telitacicept showed more stable renal function preservation, contrasting with fluctuating eGFR in the IS group. Following an increase in the first 3 months, the eGFR of the IS group declined rapidly in the final 3 months-a pattern partially attributed to the higher infection burden (75% of which occurred in month 3–6), known to accelerate IgAN progression (30). Additionally, He et al. observed an eGFR improvement only in the Telitacicept group at 3 months, while the MMF group showed a decline (14). We posit that this discrepancy may be attributed to differences in baseline pathological characteristics between the groups. Specifically, the proportion of patients with active inflammatory lesions (as indicated by M1 or E1 scores) was not only lower in their MMF group compared to their Telitacicept group but was also substantially lower than the proportions observed in both treatment groups within our cohort. This underlying imbalance could predispose their MMF group to a more pronounced eGFR decline, explaining the divergent early results. Attributing to significantly greater glucocorticoids exposure (*p* < 0.001), the IS group exhibited increased incidence of not only infections, but also metabolic disorders, including hyperglycemia, hyperuricemia, osteoporosis, and skin acne. Although with several reports of injection site reactions, the overall safety performance of telitacicept group was superior than IS group. For the treatment response, the RR of telitacicept group was significantly higher than IS group, while the differences diminished by 6 months, with no difference in final RR and no between-group differences in trajectories over 6 months. This temporal response pattern may be attributed to the distinct mechanisms of action and kinetics of the treatments. The initial superiority of telitacicept likely stems from its rapid, targeted suppression of pathogenic antibody production. In contrast, conventional immunosuppressants may exhibit a more delayed onset of their full therapeutic effect. The observed convergence at the later time point suggests that the initial kinetic advantage of a targeted therapy may eventually align with the effects of broader immunosuppression, potentially reflecting a plateau in clinical response dictated by the slower pace of underlying renal repair. Furthermore, inherent limitations of the real-world study design, including unmeasured confounding factors and potential biases introduced by patient attrition over the extended follow-up period, must be considered as alternative explanations for the observed outcome dynamics.

This study had several limitations. First, the relatively small sample size meant that this preliminary study power was not sufficient and the study was open to both type 1 and type 2 statistical errors. The fact that some patients were lost to follow-up had an impact on the study results. Second, due to the retrospective observational nature of the study design, we could not preclude unrecognized factors that confounded the results, such as life-style modification and oral medication adherence, which may be important factors in our population and assessment. As for the direct comparison between the initial and alternative cohorts mixes different lines of therapy and disease duration, introducing confounding bias and imbalances in prior exposure. And more robust methodologies, such as within-cohort (pre- versus post-) comparison for the alternative group or a landmark target trial, would account for these differences. Although implementing these ideal designs in a real-world setting patients present significant challenge, we acknowledge all limitations and risk of bias inherent in the study design. Third, the follow-up period was too short and the power of the study was not sufficient for definite conclusions. Fourth, the single-center retrospective nature of the study limits the relevance of the result, and we were unable to generalize the findings to different populations. Last, this study is limited by its propensity-score matching approach. Although separate pairwise matching was employed to address distinct clinical questions within our sample size constraints, the post-matching SMDs indicated that residual imbalance persisted for some covariates. This, combined with the inability to perform a more rigorous three-way (1:1:1) comparison, may have impacted the balance of covariates across all groups and potentially introduced unmeasured confounding. To validate our results, future large-scale prospective studies involving multiple centers are needed.

## Conclusion

In summary, our preliminary study provided evidence supporting telitacicept as an effective therapeutic option for IgAN patients at high risk of progressive kidney function loss, contributing to reduction in proteinuria and preservation in renal function, and the therapeutic benefits were more pronounced when used as initial therapy. Furthermore, telitacicept demonstrated a favorable tolerability profile. These results suggest that early intervention with telitacicept may offer better therapeutic benefits for high-risk IgAN patients.

## Data Availability

The data underlying this article will be available upon reasonable request to the corresponding author.
