# Supplementary material for: Preliminary analysis of short-term real-world outcomes of telitacicept in high-risk IgA nephropathy
Source: Front Med (Lausanne). 2025 Dec 5;12:1698028. doi: 10.3389/fmed.2025.1698028 (PMC12714628; doi:10.3389/fmed.2025.1698028)
Supplement: Supplementary file 1 [file Data_Sheet_1.docx]

**Short-term Real-world Outcomes of Telitacicept in High-Risk IgA Nephropathy**

Ying Ma^1, †^, Jing Han^2, †^, Huixian Li^1^, Xiao Yu^3^, Ping Lan^1^, Xinfang Xie^1^, Wanhong Lu^1, *^, Jiping Sun^1, *^

^1^ Department of Nephrology, Kidney Hospital, The First Affiliated Hospital of Xi’an Jiaotong University, Xian, China

^2^ Department of Nephrology, Affiliated Hospital of Northwest University, Xi’an No.3 Hospital, Xi’an, China

^3^ Department of Nephrology, Shaanxi Provincial Hospital of Traditional Chinese Medicine, Xi’an, China

^†^These authors contributed equally to this work

^*^**Corresponding authors**: Wanhong Lu ([luwanhong@xjtu.edu.cn](mailto:luwanhong@xjtu.edu.cn)), Jiping Sun✉ ([jipingsundwy@126.com](mailto:jipingsundwy@126.com))

**Key words**

IgA nephropathy, telitacicept, effectiveness, safety, real-world cohort study

**1. Distribution of propensity scores in PSM**

**Supplementary Table 1. Distribution of Propensity Scores in Treatment and Control Groups Before and After PSM Matching**

|  | **PSM between**  **initial telitacicept group and initial IS group** | | | **PSM between**  **initial and alternative telitacicept group** | | |
| --- | --- | --- | --- | --- | --- | --- |
| **Group** | **Initial telitacicept group** | **Initial IS group (before matching)** | **Initial IS group (after matching)** | **Initial telitacicept group** | **Alternative telitacicept group (before matching)** | **Alternative telitacicept group (after matching)** |
| **Sample size** | 30 | 73 | 30 | 30 | 35 | 30 |
| **Mean** | 0.3016 | 0.2870 | 0.2989 | 0.4637 | 0.4597 | 0.4647 |
| **Standard deviation** | 0.05090 | 0.05874 | 0.04935 | 0.03028 | 0.03291 | 0.03282 |
| **Minimum** | 0.21 | 0.18 | 0.20 | 0.41 | 0.42 | 0.42 |
| **Lower quartile** | 0.2622 | 0.2455 | 0.2606 | 0.4301 | 0.4314 | 0.4339 |
| **Median** | 0.2986 | 0.2854 | 0.2956 | 0.4693 | 0.4551 | 0.4597 |
| **Upper quartile** | 0.3355 | 0.3449 | 0.3438 | 0.4865 | 0.4893 | 0.4939 |
| **Maximum** | 0.39 | 0.40 | 0.39 | 0.52 | 0.53 | 0.53 |

**2. Sensitivity analysis of PSM**

**Supplementary Table 2. Adjusted analysis on the matched cohort by logistic regression model**

| **Telitacicept treatment** | **OR** | **95% CI** | **P value** |
| --- | --- | --- | --- |
| Unadjusted Model | 3.824 | 1.150-12.713 | 0.029 |
| Adjusted Model 1* | 3.866 | 1.119-13.350 | 0.033 |
| Adjusted Model 2* | 4.085 | 1.155-14.450 | 0.029 |

***** Given the sample size of the matched cohort, we prioritized adjustment for clinically established strong prognostic factors for IgAN progression, which included baseline proteinuria, eGFR, and the use of RAASi in Model 1, and baseline proteinuria, eGFR, the use of RAASi and SGLT2i in Model 2.

**3. Robustness analysis of PSM**

To ensure methodological consistency, the identical set of confounders specified for propensity-score matching (PSM) was retained in the subsequent inverse probability of treatment weighting (IPTW). After weighting, excellent covariate balance was attained: absolute standardized mean differences (SMDs) were < 0.05 for both proteinuria (SMD=0.0068) and eGFR (SMD=0.0337), well below the conventional 0.10 threshold (Table 1). Effective sample sizes were only minimally attenuated—72, 30 and 33 in the Initial IS, Initial telitacicept and Alternative telitacicept groups, respectively—indicating well-distributed weights and negligible information loss.

**
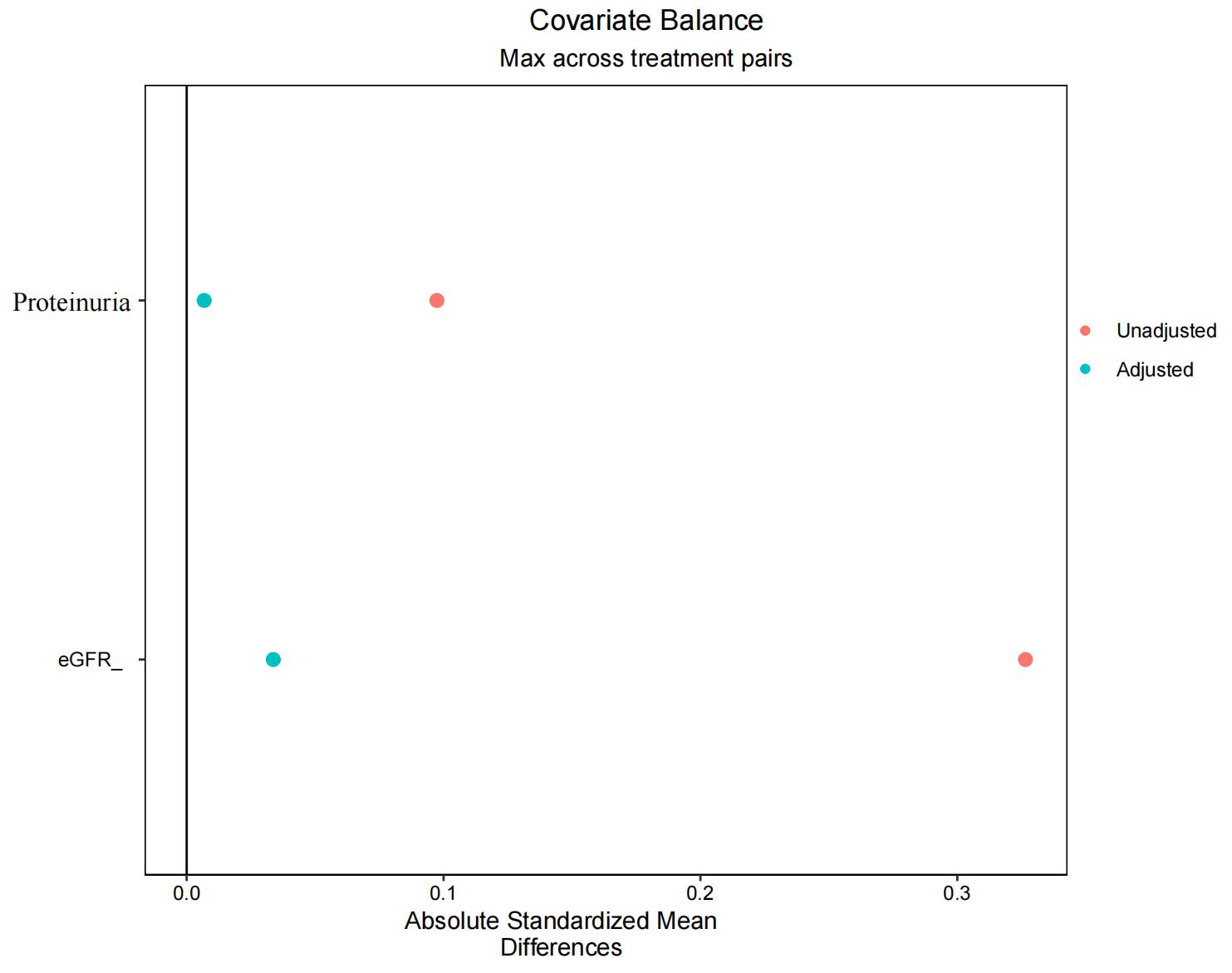
**

**Supplementary Figure 1. Love plot of standardized mean differences (SMDs) before and after inverse probability of treatment weighting (IPTW).**

Using IPTW with the Initial IS group as reference, a population-wide Initial telitacicept strategy was associated with significantly higher odds of renal response (RR) at month 3 (OR=3.457, 95% CI 1.18-10.15, P =0.024, Supplementary Figure 2).

**
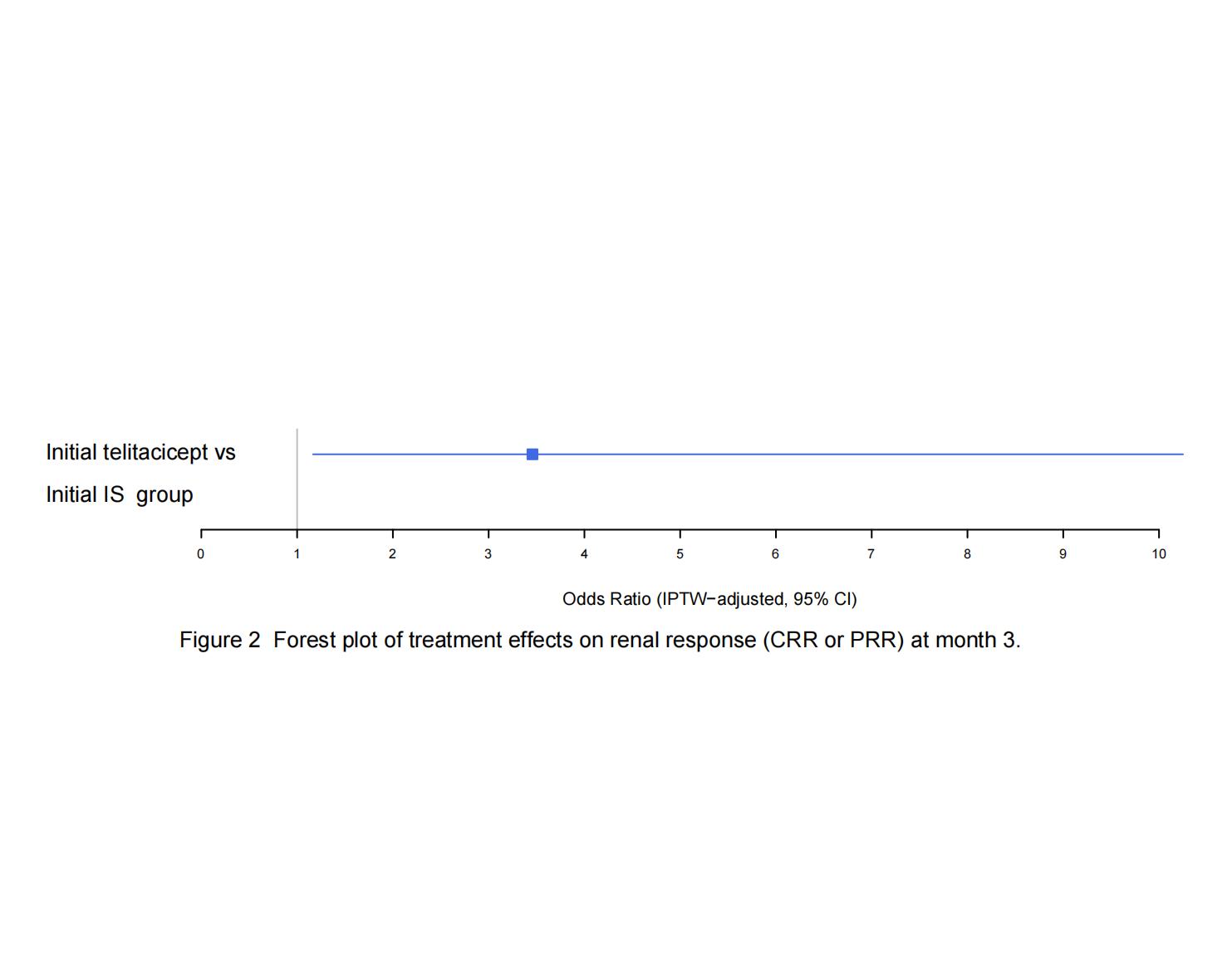
**

**Supplementary Figure 2. Forest plot of treatment effects on renal response (RR) at 3 months**

**4. Detailed changes in renal parameters during follow-up.**

**Supplementary Table 3. Changes in Follow-up Parameters in High-Risk IgA Nephropathy**

|  | **Initiative IS** | **Initiative telitacicept** | **Alternative telitacicept** | **P1 value** | **P2 value** |
| --- | --- | --- | --- | --- | --- |
| **Month3** | N=30 | N=30 | N=30 |  |  |
| SBP | 124.2±14.9 | 123.6±13.9 | 123.6±14.5 | 0.873 | 0.993 |
| DBP | 82.0±11.1 | 80.2±11.3 | 81.6±11.3 | 0.543 | 0.641 |
| Proteinuria, g/ day | 0.99(0.53,1.90) | 0.58(0.31,1.04) | 1.00(0.70,1.78) | 0.062 | 0.005 |
| Change in proteinuria from baseline, g/day | -1.15(-1.98,-0.26) | -1.47(-2.89,-0.95) | -0.88(-1.58,-0.40) | 0.081 | 0.058 |
| Percentage change in proteinuria from baseline, % | -48.1(-86.0,-20.7) | -79.7(-86.4,-51.5) | -45.8(-66.5,-29.7) | 0.065 | 0.002 |
| eGFR, ml/min/1.73m^2^ | 82.2(65.6,109.0) | 75.2(58.0,110.4) | 79.2(52.3,116.4) | 0.352 | 0.668 |
| Change in eGFR from baseline, ml/min/1.73m^2^ | 8.5(-0.5,23.7) | 3.6(-5.0,10.2) | 3.1(0.3,9.6) | 0.088 | 0.712 |
| Percentage change in eGFR from baseline, % | 9.9(-0.6,40.8) | 3.8(-8.4,21.7) | 5.7(0.6,18.2) | 0.141 | 0.802 |
| **Month 6** | N=29 | N=21 | N=16 |  |  |
| SBP | 121.3±10.7 | 119.2±13.1 | 121.1±9.6 | 0.537 | 0.609 |
| DBP | 79.2±9.5 | 79.7±11.0 | 78.3±10.1 | 0.880 | 0.699 |
| Proteinuria, g/day | 0.53(0.29,0.98) | 0.47(0.20,1.22) | 0.89(0.52,2.03) | 0.637 | 0.059 |
| Change in proteinuria from baseline, g/day | -1.23(-2.49,-0.66) | -1.54(-2.76,-0.92) | -1.07(-4.42,-0.53) | 0.596 | 0.668 |
| Percentage change in proteinuria from baseline, % | -72.8(-87.8,-42.0) | -82.8(-92.2,-56.2) | -62.6(-77.9,-41.3) | 0.510 | 0.075 |
| eGFR, ml/min/1.73m^2^ | 79.4(55.5,105.8) | 75.6(60.3,108.3) | 72.5(54.1,101.7) | 0.883 | 0.668 |
| Change in eGFR from baseline, ml/min/1.73m^2^ | 5.4(-8.9,19.3) | 1.3(-6.2,17.1) | 1.3(-8.2,7.4) | 0.930 | 0.327 |
| Percentage change in eGFR from baseline, % | 5.7(-10.2,38.0) | 2.7(-7.0,29.4) | 1.6(-10.2,16.9) | 0.776 | 0.425 |
